# Adverse selection and health insurance decisions of young migrant workers: An empirical study in China

**DOI:** 10.3389/fpubh.2023.1084133

**Published:** 2023-03-07

**Authors:** Hongbo Wang, Xi Gong

**Affiliations:** School of Public Administration and Policy, Renmin University of China, Beijing, China

**Keywords:** adverse selection, mobility distance, young migrant workers, health insurance, settlement intention

## Abstract

Using data from the China Migrants Dynamic Survey (CMDS) in 2017, this study assessed adverse selection and the impact of mobility factors on adverse selection by analyzing two samples of young migrant workers. The results of the sample analysis showed that young migrant workers with higher health risks were more inclined to enroll in health insurance, indicating the presence of adverse selection. Mobility distance and settle intention have a heterogeneous effect on adverse selection, with young workers who migrate inter-provincially and intend to settle down being more susceptible. The analysis of the insured samples showed that the phenomenon of adverse selection was also evident in the choice of health insurance, with individuals with higher risks preferring Urban Employee Basic Medical Insurance (UEBMI), which has better financial coverage and benefits compared to Rural Residents' Basic Medical Insurance (URRBMI). The heterogeneity test confirmed that mobility distance plays a role in determining the likelihood of adverse selection, with inter-city and inter-province young migrant workers being more likely to show adverse selection.

## 1. Introduction

Although the Chinese government has established a comprehensive health insurance system for all its citizens, the health insurance system is still fragmented (1). At present, China's social health insurance system comprises two components: Urban Employee Basic Medical Insurance (hereafter referred to as UEBMI), which is mandatory for formal sector workers, and Urban and Rural Residents' Basic Medical Insurance (hereafter referred to as URRBMI), which mainly serves as voluntary health insurance for unemployed residents. As they cover different populations, UEBMI and URRBMI have distinct institutional frameworks and financing systems. UEBMI has been established through an employment contract between employees and employers who are required to contribute to the monthly insurance premium. On average, the premium for UEBMI is 8% of an employee's payroll, with the employee responsible for 2% and the employer responsible for 6% (2). Eligibility for URRBMI is associated with the household registration system in China, and the contributions to URRBMI come from residents' lower fixed premium and a government subsidy, which accounts for approximately two-thirds of the total premium. In 2021, the number of people covered by URRBMI was approximately one billion, which is almost three times the number covered by UEBMI.^1^ However, there is a significant discrepancy in benefits between the two programs and regions, with UEBMI offering higher benefits due to its greater financial capacity (1). In addition, a large number of employees in informal jobs can freely choose the type of health insurance in which they want to enroll. The Chinese government expects more informal sector employees to choose UEBMI, as its larger funds allow for better distribution. However, China's universal health insurance coverage has faced many challenges in recent years.

Due to the difficulty of collecting statistics, the 95% participation rate announced by the government is not accurate. Some studies indicated that the proportion of the noninsured population in China may be up to 10% (3). Reverse selection may occur due to voluntary participation in URRBMI and the lack of supervision in some regions for UEBMI. In contrast, a larger number of individuals participate in URRBMI due to its lower premium and government subsidies. As a result, many informal sector employees may leave UEBMI for URRBMI, including some formal sector employees (4), leading to another form of adverse selection. Therefore, the study of adverse selection is crucial for the success of China's universal health insurance coverage.

With the rise of urbanization in China, a large number of migrant workers are moving from rural areas to cities for work (5). In 2017, according to the CMDS, nearly 47% of all migrants were young migrants who did not have higher education and were unable to secure jobs in the formal sector (6, 7). The number of inter-provincial migrant workers reached one million in 2019, with a total of 291 million migrant workers in the country (8, 9). The CMDS survey in 2017 indicated that only 25% of young migrant workers secured a formal job with a legal labor contract. Therefore, they have the freedom to decide whether to participate in health insurance and choose their preferred insurance type. As the premium rate of URRBMI is much lower than that of UEBMI, the majority of young migrant workers prefer to choose URRBMI in their hometown rather than UEBMI in the workplace. This preference will be more evident in the high enrollment costs caused by mobility. Young migrant workers in good health may even be able to forego health insurance. Most young migrant workers who secure a formal job usually work for medium or small enterprises. To reduce total costs, employers tend to avoid social insurance premiums and may not provide health insurance to migrant workers (9, 10). Therefore, some migrants with formal work may choose to discontinue their health insurance coverage or remain covered under the URRBMI in their hometown. Based on the data from the CMDS survey conducted in 2017, the proportion of uninsured migrant workers reached 7.26%, while only 16.38% of insured workers chose UEBMI. This finding highlights the presence of adverse selection among migrant workers, especially the young ones, with mobility as a possible contributing factor. This study aimed to empirically analyze the occurrence of adverse selection in health insurance among young migrant workers to explore the impact of mobility distance and settlement intention on adverse selection. For social health insurance funds, young migrant workers are a good risk. Understanding the motivations behind the health insurance choices made by young migrant workers will contribute to increasing health insurance coverage, especially UEBMI coverage.

Adverse selection plays an important role in the decision to buy a health insurance plan. In the insurance market, adverse selection indicates that high-risk groups are more willing to buy insurance or choose high-benefit insurance, while low-risk groups tend to refuse insurance and choose low-benefit insurance (11, 12). As the number of high-risk people increases, insurance costs will rise, and insurers will have to raise the premium, which will further lead to a crowding-out effect on the low-risk population and eventually make the insurance system unsustainable (13). Many scholars found that there exists adverse selection in China's basic health insurance system. Zang et al. (14) found that, among the urban population not covered by UEBMI, individuals with poor health status are more inclined to participate in URRBMI. Zhu and Peng (15) found the presence of adverse selection during the early stages of the implementation of URRBMI in China. Farmers who were older, had less education, earned less money, and were not migratory workers were more willing to enroll in URRBMI. The empirical study on the insurance enrollment of informal sector employees also found that individuals with high health risks are more likely to participate in basic health insurance and choose UEBMI (16). As young migrant workers have the freedom to decide whether to participate in health insurance and which health insurance to choose, there may be widespread adverse selection. The unwillingness to participate in health insurance or the preference to choose URRBMI due to adverse selection is detrimental to the social health insurance market and public health policy in China. First, it is not conducive to achieving universal health coverage if the number of uninsured migrant workers keeps growing. Without the protection of social health insurance, uninsured migrants are exposed to disease risks. Second, with more and more migrant workers choosing URRBMI instead of participating in UEBMI, the government has to bear a heavier financial subsidy burden, which has been a main challenge for the Chinese government in recent years (17). Finally, as the government plans to integrate both URBMI and URRBMI into one system with improved benefits, the decrease in the number of UEBMI participants as a result of adverse selection will further fragment the insurance system.

However, current literature has not sufficiently explored the impact of mobility on adverse selection among young migrant workers in China. Because of differences in mobility distance and settlement intentions, young migrant workers are different types of individuals for the insurance market and face different enrollment costs. On the one hand, mobility distance and settlement intentions may directly affect the health insurance decisions of young migrant workers and determine whether they choose to be insured and which kind of health insurance they prefer. On the other hand, these factors may also have heterogeneous effects on adverse selection, leading to varying levels of the likelihood of adverse selection among young migrant workers with different levels of mobility distance and settlement intentions. Based on these assumptions and combining three elements—adverse selection, mobility distance, and settlement intention—this study focuses on these three specific issues for migrant workers' insurance decisions. First, is there an adverse selection phenomenon when young migrant workers consider participating in health insurance, and which type of health insurance are they willing to choose? Second, will mobility distance and settlement intentions have a significant impact on the health insurance decision of young migrant workers? Last, will the different parameters of mobility distance and settlement intention of young migrant workers affect the possibility of adverse selection?

## 2. Theoretical basis

After the 1870s, more and more scholars began to pay attention to the issue of adverse selection in the health insurance market and used the data from the insurance market to conduct empirical studies to examine its presence (18–20). Rothschild and Stiglitz (21) examined the presence of adverse selection in the health insurance market. The authors established an asymmetric information model to test for adverse selection and found a positive correlation between insurance coverage and post-event loss. This study laid the foundation for future studies in the field. Chiappori and Salanie (22) summed up three general conclusions from adverse selection research in a competitive environment: First, the observed agent is faced with different insurance contract choices before they experience adverse selection. Second, contracts with higher protection levels are sold at higher prices. Third, the contract with the highest benefits is chosen by agents with the highest risk. These conclusions support the conventional method of testing for adverse selection in the insurance market. If the insurer provides insurance products at different prices based on the level of benefits, then the concept of adverse selection suggests that high-risk individuals are more inclined to choose products with greater benefits. When the insurance company offers only one insurance product at the same price, adverse selection implies that high-risk individuals are more likely to buy insurance products than low-risk individuals (18). Based on this theory, as the young migrant workers in China have the freedom to decide whether to opt for social health insurance and which social health insurance to buy, the chances of adverse selection occurring greatly increase. Individuals with higher health risks are more likely to opt for basic health insurance and choose UEBMI.

In addition, many studies have shown that consumer heterogeneity has an impact on adverse selection (23–25). Therefore, the heterogeneity of consumers affects not only their willingness to enroll in health insurance and their likelihood of experiencing risk but also the probability of adverse selection in the insurance market. As an important factor contributing to heterogeneity among young migrant workers (9), mobility distance and settlement intentions may also affect their insurance decisions. With an increase in mobility distance, living costs and medical care costs will rise, leading to a decrease in the willingness to enroll in insurance (26). Moreover, if young migrant workers leave the city in which they work, their welfare investment in the workplace will be reduced, and unnecessary insurance enrollment procedures can be avoided as much as possible, which will also reduce their willingness to participate in health insurance (27). Moreover, the decision of young migrant workers to choose either UEBMI at their place of employment or URRBMi at their place of registered residence also impacts their medical expenses (28). On the other hand, choosing URRBMI at their registered residence usually involves traveling back home for medical services or receiving a lower reimbursement rate when they fall ill, leading to higher medical expenses. Moreover, varying mobility distances and settlement intentions of young migrant workers also cause different life issues, such as living costs and life planning, which in turn affect the possibility of adverse selection.

## 3. Methods

### 3.1. Data sources

The cross-sectional data used in this study were sourced from the 2017 wave of the China Migrants Dynamic Survey (CMDS: https://www.chinaldrk.org.cn/wjw/#/home). The CMDS is an annual, nationwide sample survey conducted by the National Health Commission of the People's Republic of China, covering 31 provinces (districts and cities) and the Xinjiang Production and Construction Corps in China. The study focuses on the status and access of migrants to public health services in China. The respondents were residents aged 15 or older who have lived outside their registered place of residence (county or city) for 1 month or more. A total of 169,989 samples were collected using stratified sampling and the multi-stage probability proportionate to size (PPS) sampling method (29). For the purpose of the study, rural registered residents, migrant workers, and those aged 16–35 years were identified as young migrant workers. After the screening and elimination of the samples without relevant variables, 31,575 effective samples remained, including the 29,400 insured samples.

### 3.2. Variables

#### 3.1.1. Dependent variables

Adverse selection refers to the phenomenon where unhealthy individuals are more willing to participate in insurance or choose insurance plans with better benefits, even when offered at a higher price, compared to those in good health or with lower risk (12). Therefore, there are two dependent variables to measure the occurrence of adverse selection. The first was the health insurance status, which was measured by the question, “Do you have health insurance?” with a binary response (0 = uninsured, 1 = insured). The second variable is the type of insurance chosen, which was measured by asking the insured participants what kind of insurance they had, with a binary response (0 = URRBMI, 1 = UEBMI). Adverse selection occurs when people with higher health risks are more likely to either participate in insurance or choose the UEBMI option.

#### 3.1.2. Independent variables

The health risk of people is a crucial independent variable to test the existence of adverse selection (30, 31). The existing literature mainly identifies three methods to measure individual health risks. The first indicator is self-rated health, which is private information that is not easily available to the insured and can better measure the future health risk of individuals (32, 33). The second approach involves the use of medical services to measure an individual's level of health risk and determining if there is a positive correlation between individual medical expenditure and insurance choice (34). The third approach is to combine subjective health evaluation with individual objective disease history and utilization of medical services to construct a health risk indicator (16). We argue that the combination of subjective and objective health information can evaluate individual health risk levels comprehensively. Therefore, we used self-rated health and individual objective disease histories to measure health risks. The original question about individual self-rated health was, “What is your health status?” The answer includes four incremental levels, and we gave it a score ranging from 1 to 4 (“unable to take care of oneself,” coded as 1, “not healthy, but can take care of oneself,” coded as 2, “generally healthy,” coded as 3, and “healthy,” coded as 4). Objective disease history information was obtained from respondents by asking questions. Specifically, the questionnaire included questions asking whether they had experienced hypertension, diabetes, diarrhea, fever, rash, jaundice, conjunctival redness, or colds in the past year. If the sample did not suffer from disease, they were given a score of one point. Otherwise, they received a score of zero. Both subjective and objective options were scored separately and combined to calculate a comprehensive health risk score. A higher score indicates a lower level of health risk.

In addition, mobility distance was categorized into three ranges (“inter-county in a city” coded as 0, “inter-city in a province” coded as 1, and “inter-province” coded as 2). Settlement intentions were measured with the question, “Do you intend to settle here for some time?” which was recorded as a dummy variable (“leave” coded as 0, “settle down” coded as 1).

#### 3.1.3. Control variables

Based on previous studies, some variables that may affect individual risks and health insurance choices were selected as control variables. These variables include age, the logarithm of income, and education level (35–37). Health level tends to decline with age, and age is one of the key variables affecting individual health risk. Income and education levels may have an impact on the choice of health insurance. Individuals with high income levels have the financial capability to afford health insurance with a higher premium. Education is a factor that impacts an individual's understanding of insurance and may also affect their choice of health insurance. In addition, marriage and communist identity, which are the characteristics that define an individual's identity, are also considered control variables.

### 3.2. Statistical analysis

Since the dependent variable is a binary variable, we used a binary probit model for analysis. The model is as follows:


$$\begin{array}{l} \text{prob }\left({\text{Y}}_{\text{ki}}=1|{\text{X}}_{\text{i}}\right)=\text{prob }\left({\alpha +\beta {\text{Health}}_{\text{i}}+\gamma {\text{ Move}}_{\text{i}}+\gamma \text{X}}_{\text{i}}+\varepsilon_{\text{i}}\right) \end{array}$$

In this equation, *Y*_*ki*_ is a discrete binary variable, and *Y*_*ki*_ = 1 indicates the probability of the young migrant workers participating in health insurance or choosing UEBMI; otherwise, *Y*_*ki*_ = 0. The values of *k* are 1 and 2, which, respectively, indicate that young migrant workers participate in health insurance and choose UEBMI. *Health*_*i*_ is the score of one's health level, and β is the parameter to be estimated when testing for adverse selection. When controlling for other variables, a negative β indicates that young migrant workers with lower scores on individual health levels are more likely to participate in health insurance or choose UEBMI; that is, the health risk of young migrant workers is positively correlated with the possibility of participating in health insurance or UEBMI, with a possibility of adverse selection. *Move*_*i*_ is the mobility characteristics of young migrant workers, including mobility distance and settlement intentions. *X*_*i*_are control variables, such as age, income, marriage, education level, and communist identity. ε_*i*_ is a random error term.

The empirical analysis of the study includes three parts. First, we analyzed the entire sample to examine the adverse selection of young migrant workers while deciding to participate in health insurance and the influence of mobility characteristics on the decision-making process. Second, we examined only insured individuals to assess the presence of adverse selection among young migrant workers while choosing the type of health insurance and the influence of mobility characteristics on the choices. Finally, the heterogeneous effect of mobility characteristics on adverse selection was also tested using grouping samples based on their mobility distance and settlement intentions.

## 4. Results

### 4.1. Descriptive statistics

Table 1 shows the characteristics of variables in both the total number of samples and the insured sample. Table 2 shows the results of the bivariate analysis. The respondents with different mobility distances and settlement intentions had significant differences in their participation in insurance and their selection of insurance types. These findings indicate that mobility characteristics may significantly affect insurance choices.

**Table 1:** Descriptive statistics of main variables.

**Variable**	**Total sample**		**Insured sample**	
	***N***	**Percentage (%)**	***N***	**Percentage (%)**
**Insured or not**				
Uninsured	2,175	6.89		
Insured	29,400	93.11		
**Insurance type**				
URRBMI			23,705	80.93
UEBMI			5,695	19.37
**Gender**				
Male	13,585	43.02	12,727	43.29
Female	17,990	56.98	16,673	56.71
**Marital status**				
Unmarried	2,660	8.42	2,333	7.94
Married	28,915	91.58	27,067	92.06
**Education level**				
Primary school and below	2,610	8.27	2,347	7.98
Junior high school	16,272	51.53	15,122	51.44
High school	8,130	25.75	7,631	25.96
University or above	4,563	14.45	4,300	14.63
**Communist or not**				
Non communist	30,650	97.07	28,532	97.05
Communist	925	2.39	868	2.95
Variable name	Mean	SD	Mean	SD
Health score	8.49	1.29	8.48	1.29
Age	29.13	3.98	29.19	3.93
Logarithm of income	8.84	0.57	8.85	0.56

**Table 2:** Bivariate analysis results.

**Variable**	**Insured or not**		**χ^2^**	***p***	**Insurance type**		**χ^2^**	***P***
	**Uninsured**	**Insured**			**URRBMI**	**UEBMI**		
**Mobility range**								
Inter-county in a city	165	4,520			4,016	504		
Inter-city in a province	539	9,067	178.15	0.000^***^	7,260	1,807	239.066	0.000^***^
Inter-province	1,471	15,813			12,429	3,384		
**Settlement intention**								
Leave	434	4,493	33.56	0.000^***^	3,974	519	207.62	0.000^***^
Settle down	1,741	24,907			19,731	5,176		

In this table, ^*^indicates *p* < 0.1, ^**^indicates *p* < 0.05, and

*** indicates *p* < 0.01, similarly for other tables.

### 4.2. Adverse selection when deciding to participate in health insurance and the effect of mobility characteristics on the decision

Table 3 shows the result of the adverse selection analysis and the effect of mobility distance and settlement intentions on the decision to participate in health insurance using the whole sample. Model 1 is a benchmark model that only includes control variables, in which age, marital status, gender, education, and the logarithm of income all have a significant influence on the decision to participate in health insurance. The participation rates keep improving with age, and the result is consistent with some previous studies. For example, Chen et al. (38) found that migrant workers under the age of 20 years had higher rates of being uninsured. Men are more likely to participate in health insurance than women, and this fact is consistent with previous studies (39). Compared to workers with primary school level education or below, those with higher education were positively correlated with higher health insurance participation rates, as reported in some studies (38). Additionally, as the logarithm of income increases, young migrant workers are found to be more likely to participate in health insurance. The results of the control variable analysis are generally consistent with the findings of previous studies. In Model 2, after controlling for other variables, the health score was found to have a negative correlation with participation in health insurance, at a significance level of 1%. This suggests that there is a positive relationship between health risk and participation in health insurance, where individuals with higher health risks are more likely to participate in health insurance. Adverse selection occurs when young migrant workers decide to participate in insurance. After taking into account mobility distance and settlement intentions, we observed that Model 3 shows that when controlling for other variables, compared with young migrant workers inter-county in a city, the possibility of participating in health insurance for young migrant workers inter-city in a province and inter-province is lower, both of which are at the 1% significant level. This shows that the extension of mobility distance reduces the probability of young migrant workers participating in health insurance. In terms of settlement intentions, compared with young migrant workers who tend to leave, the settle-down group is more likely to participate in health insurance. At the same time, although mobility factors are controlled, there is still a positive correlation between the health risk of young migrant workers and their probability of participating in health insurance, and an adverse selection effect still exists. The aforementioned analysis revealed that, from the whole sample, adverse selection occurs when young migrant workers decide to participate in health insurance. Mobility distance and settlement intentions were found to have a significant impact on the decision-making process.

**Table 3:** Regression analysis results of whether or not to participate in health insurance.

**Variable**	**Whether or not to participate in health insurance**		
	**Model 1**	**Model 2**	**Model 3**
Age	0.020^***^	0.020^***^	0.017^***^
	(0.003)	(0.003)	(0.003)
**Marital status (unmarried)**			
Married	0.268^***^	0.269^***^	0.287^***^
	(0.039)	(0.039)	(0.039)
**Gender (male)**			
Female	−0.061^***^	−0.064^***^	−0.074^***^
	(0.023)	(0.023)	(0.023)
**Education (primary school and below)**			
Junior high school	0.216^***^	0.220^***^	0.211^***^
	(0.037)	(0.037)	(0.037)
High school	0.308^***^	0.312^***^	0.274^***^
	(0.041)	(0.041)	(0.041)
University or above	0.333^***^	0.336^***^	0.278^***^
	(0.047)	(0.047)	(0.047)
**Communist or not (not)**			
Communist	−0.024	−0.030	−0.032
	(0.068)	(0.068)	(0.069)
Logarithm of income	0.039^**^	0.038^**^	0.065^***^
	(0.018)	(0.018)	(0.018)
Health score		−0.025^***^	−0.017^**^
		(0.008)	(0.008)
**Mobility distance (inter-county in a city)**			
Inter-city in a province			−0.218^***^
			(0.041)
Inter-province			−0.432^***^
			(0.038)
**Settlement intention (leave)**			
Settle down			0.093^***^
			(0.029)
Intercept term	0.118	0.334^*^	0.378^*^
	(0.179)	(0.193)	(0.194)
*N*	31,575	31,575	31,575
Pseudo *R^2^*	0.016	0.016	0.028

The items in parentheses in the variable column represent reference groups and parentheses in the model column represent standard errors.

* *p* < 0.1,

** *p* < 0.05,

*** *p* < 0.01.

### 4.3. The presence of adverse selection when choosing a health insurance plan and the effect of mobility characteristics on the choice

Table 4 depicts the results of the analysis of adverse selection in the choice of health insurance type and the influence of mobility characteristics on the choice. Model 4 is a benchmark model that only includes control variables. When controlling for other variables, each variable, such as age, gender, education, communist identity, and income logarithm, played a significant role. After considering health scores in Model 5, the results revealed that the health score of young migrant workers was significantly negatively correlated with the decision to opt for UEBMI at the 1% significant level; that is, the health risk is positively correlated with the possibility of opting for UEBMI. Therefore, adverse selection occurs when young migrant workers choose their health insurance type, and individuals with lower health levels are more inclined to opt for UEBMI. Model 6 considers mobility distance and settlement intentions. Controlling for other variables, we found that the degree of health risks was still positively correlated with the possibility of participating in UEBMI at the 1% significant level, and adverse selection persisted. At the same time, mobility distance and settlement intention had a significant impact on young migrant workers' insurance choices. Compared with young migrant workers inter-county in a city, migrant workers inter-city in a province and inter-province were more likely to participate in UEBMI. Compared with young migrant workers who tended to leave, individuals planning to settle down were more likely to participate in UEBMI. The analysis shows that adverse selection exists when young migrant workers chose health insurance types. People with higher health levels were more likely to participate in URRBMI, which has lower insurance premiums and lower benefit levels, while people with higher health risk levels were more likely to participate in UEBMI, which has higher insurance premiums and higher benefit levels. At the same time, mobility distance and settlement intention had a significant impact on the choice of young migrant workers' insurance type, which indicates that it is necessary to analyze whether different mobility distances and settlement intentions affect the possibility of adverse selection.

**Table 4:** Regression analysis results of health insurance type choice.

**Variable**	**Whether or not to participate in UEBMI**		
	**Model 4**	**Model 5**	**Model 6**
Age	0.018^***^	0.018^***^	0.020^***^
	(0.003)	(0.003)	(0.003)
**Marital status (unmarried)**			
Married	0.075^**^	0.074^**^	0.055
	(0.038)	(0.038)	(0.038)
**Gender (men)**			
Women	−0.062^***^	−0.063^***^	−0.054^***^
	(0.018)	(0.018)	(0.018)
**Education (primary school and below)**			
Junior high school	0.388^***^	0.390^***^	0.394^***^
	(0.045)	(0.045)	(0.045)
High school	0.732^***^	0.735^***^	0.764^***^
	(0.046)	(0.046)	(0.047)
University or above	1.398^***^	1.400^***^	1.437^***^
	(0.048)	(0.048)	(0.048)
**Communist or not (not)**			
Communist	0.219^***^	0.215^***^	0.226^***^
	(0.047)	(0.047)	(0.048)
Logarithm of income	0.407^***^	0.405^***^	0.348^***^
	(0.018)	(0.018)	(0.018)
Health score		−0.030^***^	−0.033^***^
		(0.007)	(0.007)
**Mobility distance (inter-county in a city)**			
Inter-city in a province			0.344^***^
			(0.031)
Inter-province			0.484^***^
			(0.029)
**Settlement intention (leave)**			
Settle down			0.249^***^
			(0.028)
Intercept term	−5.715^***^	−5.449^***^	−5.569^***^
	(0.176)	(0.186)	(0.188)
*N*	29,400	29,400	29,400
Pseudo *R^2^*	0.117	0.117	0.129

* *p* < 0.1,

** *p* < 0.05,

*** *p* < 0.01.

### 4.4. Endogenous analysis and robustness test

#### 4.4.1. The endogenous analysis and IV-Probit solutions

In the above analysis, there may be a reverse causality between settlement intentions and the insurance participation behaviors of young migrant workers, which suggests that settlement intentions may be an endogenous variable. Specifically, according to the aforementioned analysis, settlement intentions affect the insurance participation behaviors of young migrant workers. Young migrant workers who tend to settle down are more likely to join the health insurance program and are more motivated to choose UEBMI. However, the decision to settle may also be influenced by insurance participation behaviors. For example, uninsured young migrant workers may prefer to leave the city where they work due to a lack of access to health services, while young migrant workers who are covered by UEBMI are more likely to settle down in their working city to continue receiving better benefits.

To address the endogenous issue, we employed the IV-Probit model for further analysis and testing. The IV-Probit model is the application of IV (instrumental variables) to the probit model. Generally, a valid IV should meet two key application conditions: “correlation restriction,” which indicates that the IV must be correlated with the potentially endogenous treatment variable, and “exclusion restriction”, indicating that the IV should have no direct effect on the dependent variable (40). According to the above restriction, we chose the “participation level of social activities” of young migrant workers in the inflow area as the IV. The participation level of social activities was measured by the question, “Did you participate in the activities of the following organizations locally last year?” The options included six types: “trade unions, volunteer associations, alumni associations, hometown associations, hometown chambers of commerce, and others”. One point was given for participating in one type of social activity, and the participation rate of social activities was the cumulative plus score. The participation rate of social activities meets the conditions for IV. First, it was correlated with the potential endogenous treatment variable (settlement intention). A high score on the participation level in social activities suggests that young migrants have been successfully assimilated into the community; therefore, they are more likely to settle down. Second, as the level of participation in social activities reflects an individual's social identity, it has no direct effect on the outcome variable (insurance participation behaviors).

The results of the two-stage estimators of the IV-Probit method are shown in Table 5. The Wald exogenous exclusion test rejects the null hypothesis, which indicates that settlement intention is an endogenous variable. Moreover, the joint test results of AR and Wald also rejected the hypothesis of a weak instrumental variable, confirming that the level of participation in social activities is an appropriate instrumental variable. Model 7 and Model 9 show the first-stage estimators, while the second-stage estimators are shown in Model 8 and Model 10. Model 8 and Model 10 display that settlement intention continues to have a significant impact on the health insurance decision-making process of young migrant workers, even after considering the instrumental variable. The estimation results are generally consistent with those of the original model.

**Table 5:** Estimation results of IV-probit model.

**Variable name**	**Modle 7**	**Modle 8**	**Modle 9**	**Modle 10**
	**Settlement intention**	**Whether or not to participate in health insurance**	**Settlement intention**	**Whether or not to participate in UEBMI**
Settlement intention		7.614^***^		9.775^***^
		(1.445)		(1.729)
Participation level of social activities	0.014^***^		0.013^***^	
	(0.003)		(0.002)	
Control variables	Controlled	Controlled	Controlled	Controlled
Intercept term	0.186^***^	−1.129^***^	0.182^***^	−7.518
	(0.035)	(0.412)	(0.037)	(0.496)
*N*	31,575	31,575	29,400	29,400
Adjust *R^2^*		0.023		0.023
Wald exogenous test		68.86^***^		197.65^***^
Wald weak IV test		27.78^***^		31.94^***^
AR weak IV test		70.53^***^		207.25^***^

* *p* < 0.1,

** *p* < 0.05,

*** *p* < 0.01.

#### 4.4.2. Robustness test

We conducted a robustness test on the model by considering a bilateral 1% tailing of income variables. Table 6 shows the results from the regression analysis after we adjusted the sample. Model 11 is the benchmark model with only control variables included; Model 12 is the regression model with a health score; and Model 13 shows the analysis results with mobility distance and settlement intentions. The direction and significance of the regression coefficient in the three models are consistent with those of the original model, indicating that adverse selection exists when young migrant workers decide to participate in health insurance. Moreover, mobility distance and settlement intentions have an impact on young migrant workers' insurance decision-making processes. The results of the regression analysis after tailoring the insured sample are shown in Models 14 and 16, and they also support the robustness of the original model and the existence of adverse selection in the choice of health insurance type by young migrant workers.

**Table 6:** Regression results after tailing of control variables (income).

**Variable**	**Whether or not to participate in health insurance**			**Whether ot not to participate in UEBMI**		
	**Modle 11**	**Modle 12**	**Modle 13**	**Modle 14**	**Modle 15**	**Modle 16**
Age	0.020^**^	0.020^**^	0.017^**^	0.018^***^	0.018^***^	0.020^***^
	(0.003)	(0.003)	(0.003)	(0.003)	(0.003)	(0.003)
**Marital status (unmarried)**						
Married	0.268^***^	0.269^***^	0.287^***^	0.075^**^	0.073^*^	0.055
	(0.039)	(0.039)	(0.039)	(0.038)	(0.038)	(0.038)
**Gender (men)**						
Women	−0.062^***^	−0.064^***^	−0.074^***^	−0.060^***^	−0.062^***^	−0.053^***^
	(0.023)	(0.023)	(0.023)	(0.018)	(0.018)	(0.018)
**Education (primary school and below)**						
Junior high school	0.217^***^	0.221^***^	0.211^***^	0.384^***^	0.386^***^	0.391^***^
	(0.037)	(0.037)	(0.037)	(0.045)	(0.045)	(0.045)
High school	0.310^***^	0.313^***^	0.273^***^	0.727^***^	0.731^***^	0.760^***^
	(0.041)	(0.041)	(0.041)	(0.046)	(0.046)	(0.047)
University or above	0.335^***^	0.338^***^	0.276^***^	1.391^***^	1.394^***^	1.431^***^
	(0.047)	(0.047)	(0.047)	(0.048)	(0.048)	(0.048)
**Communist or not (not)**						
Communist	−0.024	−0.030	−0.032	0.219^***^	0.214^***^	0.225^***^
	(0.068)	(0.068)	(0.069)	(0.047)	(0.047)	(0.048)
Logarithm of income	0.037^*^	0.079	0.174^***^	0.434^***^	0.432^***^	0.372^***^
	(0.022)	(0.022)	(0.023)	(0.018)	(0.018)	(0.019)
Health score		−0.025^***^	−0.017^**^		−0.030^***^	−0.033^***^
		(0.008)	(0.008)		(0.007)	(0.007)
**Mobility distance (inter-county in a city)**						
Inter-city in a province			−0.219^***^			0.342^***^
			(0.041)			(0.031)
Inter-province			−0.434^***^			0.480^***^
			(0.038)			(0.030)
**Settlement intention (leave)**						
Settle down			0.092^***^			0.246^***^
			(0.029)			(0.028)
Intercept term	0.131	0.350	0.296	−5.951^***^	−5.687^***^	−5.773^***^
	(0.131)	(0.350)	(0.296)	(0.181)	(0.191)	(0.194)
*N*	31,575	31,575	31,575	29,400	29,400	29,400
Pseudo *R*^2^	0.016	0.016	0.028	0.108	0.109	0.120

* *p* < 0.1,

** *p* < 0.05,

*** *p* < 0.01.

### 4.5. The heterogeneous effect of mobility characteristics on adverse selection

To further analyze the heterogeneous effect of mobility characteristics on adverse selection, we performed a group subdivision test on the young migrant workers according to their mobility distance and settlement intention.

First, using the whole sample, we analyzed whether the adverse selection experienced by young migrant workers when participating in health insurance is affected by mobility distance and settlement intention. Table 7 shows the regression results after grouping young migrant workers based on their different mobility distances. It was found that there is only a negative correlation between the health score of young migrant workers who move between provinces and their likelihood of being insured. Moreover, there is a positive correlation between health risks and the possibility of participating in health insurance at the 5% significant level. After grouping young migrant workers based on different settlement intentions, it was found that the health risk of young migrant workers who tend to settle is positively correlated with the possibility of participating in health insurance at the 5% significance level. This finding shows that young migrant workers who have differentmobility distances and settlement intentions exhibit different adverse selection behaviors in their decision to participate in health insurance. The inter-province young migrant workers and those who tend to settle down are more likely to have adverse selection behavior.

**Table 7:** Heterogeneous effect of mobility distance and settlement intention on adverse selection in health insurance participation.

**Variable**	**Whether or not to participate in health insurance**				
	**Inter-county in a city**	**Inter-city in a province**	**Inter-province**	**Leave**	**Settle down**
Health score	0.016	−0.013	−0.025^**^	0.007	−0.024^**^
	(0.027)	(0.016)	(0.011)	(0.020)	(0.009)
Control variables	Controlled	Controlled	Controlled	Controlled	Controlled
Intercept term	0.738^*^	0.661^***^	0.555^***^	0.937^***^	1.005^***^
	(0.396)	(0.227)	(0.145)	(0.269)	(0.134)
*N*	4,685	9,606	17,284	4,927	26,648
Pseudo *R^2^*	0.023	0.017	0.017	0.027	0.026

* *p* < 0.1,

** *p* < 0.05,

*** *p* < 0.01.

Second, using the insured sample, we analyzed whether the occurrence of adverse selection is related to mobility distance and settlement intentions when making a decision regarding the type of health insurance to buy. Table 8 shows that the health score of young workers migrating inter-city in a province and inter-province are negatively correlated with their participation in UEBMI at the 1% significant level. In other words, the level of health risk of these two types of young migrant workers is positively correlated with the possibility of participating in UEBMI, and there is no adverse selection among young migrant workers from inter-county in a city. Meanwhile, the results of grouping by settlement intentions show that young migrant workers with higher health risks are more likely to participate in UEBMI, regardless of whether they leave or settle. In other words, when choosing a health insurance plan, mobility distance has a heterogeneous effect on the adverse selection behavior of young migrant workers. Young migrant workers who move between cities within a province or between provinces are more likely to are more likely to exhibit adverse selection behavior, while their settlement intentions do not have a heterogeneous effect on their behavior.

**Table 8:** Heterogeneous effect of migration distance and settlement intention on adverse selection in insured sample.

**Variable**	**Whether or not to participate in UEBMI**				
	**Inter-county in a city**	**Inter-city in a province**	**Inter-province**	**Leave**	**Settle down**
Health level	−0.030	−0.055^***^	−0.027^***^	−0.044^**^	−0.035^***^
	(0.021)	(0.012)	(0.009)	(0.020)	(0.007)
Control variable	Controlled	Controlled	Controlled	Controlled	Controlled
Intercept term	−2.657^***^	−2.200^***^	−2.182^***^	−2.397^***^	−2.443^***^
	(0.358)	(0.200)	(0.138)	(0.295)	(0.117)
*N*	4,520	9,067	15,813	4,93	24,907
Pseudo *R^2^*	0.137	0.116	0.099	0.089	0.112

* *p* < 0.1,

** *p* < 0.05,

*** *p* < 0.01.

The results of the grouping test show that mobility distance and settlement intentions have a heterogeneous influence on the adverse selection of young migrant workers in health insurance participation. When participating in health insurance, young migrant workers who move farther away and tend to settle down are more likely to exhibit adverse selection behavior. When choosing a health insurance plan, although settlement intentions do not have a heterogeneous effect, young migrant workers who move farther away are more likely to have adverse selection behavior.

## 5. Discussion

Using the survey data from the CMDS (2017)^2^ and the probit model, this study thoroughly analyzed the impact of adverse selection on young migrant workers' health insurance participation and their choice of health insurance type. More importantly, this study considered mobility characteristics and tested the effect of different mobility characteristics on the occurrence of adverse selection among young migrant workers. This study's empirical analysis revealed the following insights: (1) In the decision-making process of young migrant workers' health insurance participation, those with higher health risks are more likely to participate in health insurance and exhibit adverse selection. Mobility distance and settlement intentions have a significant impact on health insurance participation. Compared with young migrant workers from inter-county in a city, those from inter-city in a province and inter-province are less likely to participate in health insurance; that is, the extension of mobility distance for young migrant workers reduces the probability of participating in health insurance. Settlement intentions have a positive and significant influence on young migrant workers' health insurance participation. Compared with young migrant workers who tend to leave, those who prefer to settle down are more likely to participate in health insurance. Moreover, mobility factors play a heterogeneous role in affecting adverse selection in health insurance decisions. Young migrant workers who migrate interprovincially and those who are inclined to settle down are more likely to exhibit adverse selection behavior. (2) The analysis of insured samples showed that adverse selection occurs when young migrant workers choose a particular type of health insurance. People with higher health risks are more likely to participate in UEBMI, which has a higher premium and improved benefits, while people with lower health risks are more inclined to opt for URRBMI, which has a lower premium and fewer benefits. Mobility distance and settlement intentions also have a significant influence on the choice of a health insurance plan. Young migrant workers who migrate inter-city or inter-province or are inclined to settle down in the local area are more likely to participate in UEBMI. The heterogeneity test showed that, when choosing a particular type of health insurance, those who migrate inter-city and inter-province are more likely to exhibit adverse selection, while settlement intentions do not have a heterogeneous effect on the occurrence of adverse selection.

This study's findings showed that the problem of adverse selection is particularly prevalent among young migrant workers when it comes to their participation in China's social health insurance system. The occurrence of adverse selection increases the systematic risk of social health insurance, and the combination of mobility factors complicates the situation, leading to a decrease in the number of insured people and reduced participation in UEBMI. This can pose challenges to the goal of universal coverage and system integration in China. These factors present obstacles to achieving universal health insurance coverage. Furthermore, adverse selection leads to a decrease in the number of participants in UEBMI, further increasing the number of participants in URRBMI. This shift from UEBMI to URRBMI puts increased pressure on the government, making the health insurance system unbalanced. As a result, achieving system integration becomes challenging. The adverse selection issue combined with mobility factors exacerbates the two major problems of insufficient coverage and an imbalanced structure in the social health insurance system, hindering young migrant workers from experiencing the full benefits of insurance.

There are several limitations to this study. First, being based on cross-sectional data, it is difficult to fully capture the influence of adverse selection over time, which requires further research using panel data. Second, limited by the available data, some important variables were omitted or suboptimal variables were used in the analysis. For example, due to the low response rate on the occupation type and employment sector type, we could not include “occupation condition” as a control variable, despite its significant influence on young migrant workers' insurance participation behaviors. Moreover, since there are no specific objective indicators to measure the health level in the original questionnaire, the validity of measuring the health of young migrant workers may have been reduced. Finally, due to the limitation of causal inference, the analysis still belongs to correlation analysis and cannot determine causal relationships between variables.

## 6. Conclusion

Although China's health insurance has already made great strides, the problem of adverse selection persists. Migrant workers, in particular, face varying degrees of adverse selection risk due to their differing mobility characteristics. This study made two theoretical contributions. First, previous studies on adverse selection in health insurance have often focused on determining the existence of adverse selection in certain countries' insurance plans. Only a few researchers examined the relationship between individual heterogeneity and adverse selection, with a focus on how demographics, such as gender, age, and race, affect adverse selection (41, 42). The study adds to the literature by exploring the relationship between adverse selection and individual social characteristics. The results provide a comprehensive understanding of the heterogeneity of adverse selection and its underlying mechanisms. Second, in the study of Chinese migrant workers and social health insurance, many scholars examined the mobility characteristics of these migrant workers or their insurance decision-making; however, few have explicitly studied the interaction between the two. This study fills this gap by combining the concepts of mobility and adverse selection to shed light on the impact of adverse selection among migrant workers, making a valuable contribution to existing research. From a practical standpoint, it is necessary for the Chinese government to address the issue of adverse selection by taking appropriate measures. First, government policies should be revised to increase migrant workers' willingness to participate in the UEBMI. For example, as the majority of migrant workers have informal jobs, their insurance premiums could be lower than those for formal employees. Second, as a number of businesses are inclined to avoid providing insurance benefits to migrant workers, stricter regulations for employers are essential. Third, the government should promote the equalization of public services so that migrant workers can also receive fair treatment in cities, which in turn will increase their willingness to settle down.

## Data availability statement

Publicly available datasets were analyzed in this study. This data can be found here: https://www.chinaldrk.org.cn/wjw/#/home.

## Author contributions

HW: conceptualization, methodology, formal analysis, and writing the manuscript. XG: formal analysis and reviewing the manuscript. All authors contributed to the article and approved the submitted version.

## Footnotes

^1^Available online at: http://www.nhsa.gov.cn/art/2022/6/8/art_7_8276.html (accessed November 20, 2022).

^2^Available online at: https://www.chinaldrk.org.cn/wjw/#/data/classify/population (accessed July 11, 2022).
